# Protective Effects of Plum on Liver and Gut Injury in Metabolic Dysfunction-Associated Fatty Liver Disease

**DOI:** 10.3390/nu16213760

**Published:** 2024-11-01

**Authors:** Ji-Su Kim, Sun-Mee Hong, Do-Kyun Kim, Young-Eun Cho

**Affiliations:** 1 Department of Food and Nutrition, Andong National University, Andong 36729, Republic of Korea; witn6265@naver.com; 2 Department of Technology Development, Marine Industry Research Institute for East Sea Rim, Uljin 36315, Republic of Korea; hongsunmee@mire.re.kr; 3 Korea Zoonosis Research Institute, Jeonbuk National University, Iksan 54531, Republic of Korea

**Keywords:** metabolic dysfunction-associated fatty liver disease (MASLD), freeze-dried plum (FDP), liver damage, gut damage

## Abstract

Metabolic dysfunction-associated fatty liver disease (MASLD), a persistent liver condition associated with metabolic syndrome, is primarily caused by excessive fructose intake and a typical Western diet. Because there is currently only one approved treatment, lifestyle and dietary interventions are crucial. This study assessed the effects of dietary intervention involving freeze-dried plum (FDP), a natural source of antioxidants containing diverse polyphenols. This study aimed to assess its potential as a protective agent against the gut–liver axis and its therapeutic effects on liver injury and gut permeability issues associated with MASLD. We indicate that 10% FDP intake restored gut barrier proteins and reduced serum endotoxin levels in the MASLD mouse models. Additionally, 10% FDP intake significantly reduced hepatic oxidative stress, lipid metabolism, and fibrosis marker levels. Interestingly, FDP intake significantly reduced the levels of inflammatory cytokine tumor necrosis factor-α and markers of liver damage, such as serum alanine aminotransferase/aspartate aminotransferase and hepatic triglycerides. These results highlight that dietary intervention with FDP that acts as a natural antioxidant may be a significant protective and therapeutic agent against liver and gut damage caused by MASLD.

## 1. Introduction

Metabolic dysfunction-associated fatty liver disease (MASLD) is a chronic liver disease associated with metabolic syndrome that occurs despite the absence of binge alcohol consumption [1,2]. It affects over 30% of the global population, with steadily increasing prevalence. MASLD is primarily caused by obesity and related diseases that are associated with high fructose intake and a Western diet [3,4]. MASLD is associated with an increased risk of fatty liver, metabolic dysfunction-associated steatohepatitis, cirrhosis, hepatocellular carcinoma, cardiovascular diseases, and certain cancer types [5]. Currently, there is only one approved drug for MASLD treatment [6], highlighting the immediate need for strategies to address the MASLD-based significant economic burden on the healthcare system.

As mentioned earlier, an increase in MASLD poses a significant economic challenge. With only one approved treatment or drug currently available, recognition, rapid diagnosis, and management of the disease are the primary preventive methods [4]. Although combination therapy with anti-lipoproteins such as resmetirom and type 2 diabetes medications have been reported to be safe, side effects due to long-term treatment require further monitoring [7,8]. Therefore, weight loss and a healthy diet are recommended to reduce the clinical and economic burden of MASLD [9]. Specifically, dietary interventions, such as consuming fruits rich in anti-inflammatory and antioxidant properties and vitamins, are crucial for reducing liver inflammation levels [10,11,12].

Fruits are important foods that help delay chronic diseases and improve health. They contain vitamins, trace minerals, dietary fiber, and various phytochemicals, which provide antioxidant effects, detoxification, immune support, cholesterol regulation, and blood pressure reduction [12,13,14].

Japanese plums (*P. salicina*) are rich in vitamins and minerals and exhibit higher antioxidant activity than many other fruits [15,16,17,18]. Plums contain anthocyanins, flavonols, and carotenoids [19] and exhibit beneficial effects against various diseases, such as obesity [20], diabetes [19], and cardiovascular diseases [21]. Current studies on inflammatory bowel disease [17], bone disease [22], and cancer [23] support the high antioxidant activity of plums. Recently, our group reported that the Japanese plums (Daeseok) exhibit a protective effect on DSS (dextran sulfate sodium)-induced acute/chronic colonic [17] or induce differentiation of osteoblasts [22].

**Figure 1:**
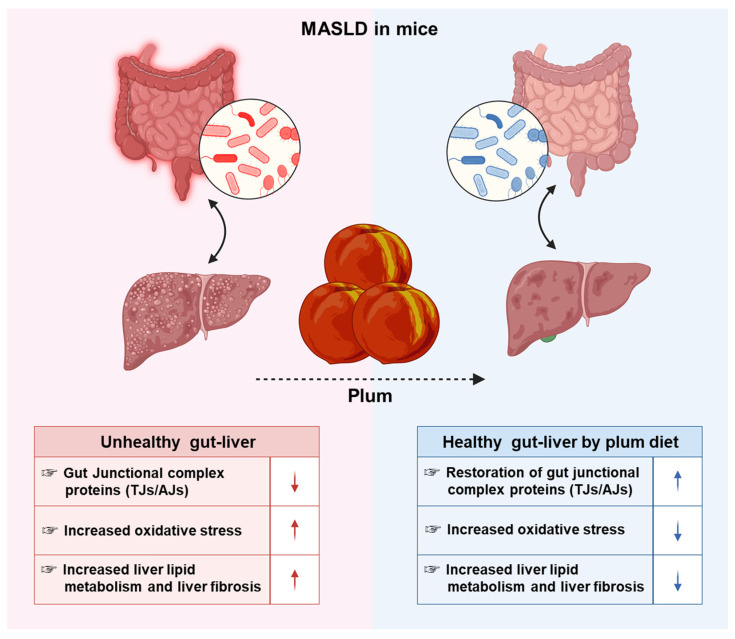
This summary highlights the protective effects of plum against oxidative stress, intestinal permeability, and liver fibrosis induced by diet high in fat, fructose, and cholesterol. The left and right illustrations indicate an increase in these parameters in the metabolic dysfunction-associated fatty liver disease mouse model and a decrease in each parameter owing to the plum diet, respectively. Upward and downward arrows also represent increase and decrease, respectively.

However, there is currently limited evidence to support studies on the dietary interventions of plums for MASLD. Therefore, this study assessed the preventive effects of plum dietary intervention through the protection of the gut–liver axis in a mouse model of MASLD induced by a high-fructose diet (Figure 1).

## 2. Materials and Methods

### 2.1. Animal Ethics and Treatment

All animal experiments were approved by the Andong University Animal Care and Use Committee (Approval No. 2021-2-0420-01-01 on 4 April 2021) and adhered to the Andong University Small Animal Testing Guidelines. All female C57BL/6J mice (6–8 weeks old) were purchased from Koatech Inc. (Pyeongtaek-si, Republic of Korea) and housed under a 12 h light–dark cycle with free access to food and water. To induce MASLD, the mice were fed a Western-style diet rich in fat, fructose, and cholesterol (FFC) (DooYeol Biotech, Seoul, Republic of Korea). Animals were randomly divided into three groups (*n* = 5 mice/group): (a) CON group: fed on standard chow; (b) FFC group: fed on FFC diet for 8 weeks; (c) FFC + 10% freeze-dried plum (FDP) group: fed on FFC diet for 8 weeks and 10% FDP diet for 5 weeks.

### 2.2. Preparation of Oriental Plum Powder

Briefly Japanese plums (Daeseok) (Uiseong Nonghyup, Uiseong-si, Republic of Korea) were grown on a farm in Uiseong, Gyeongsangbuk-do, and harvested at their peak in July 2020. After harvest, the plums went through a thorough washing process. The plum powder was produced using the method described in a prior study [17]. They were freeze-dried, powdered, and left frozen until used in the AIN-76A synthetic diet (DooYeol Biotech, Seoul, Republic of Korea) at a concentration of 10% based on the previous report.

### 2.3. The Oral Glucose Tolerance Test

After 5 weeks of dietary intervention with 10% FDP, blood was collected from the tails of all mice to assess baseline glucose and insulin levels. For the oral glucose tolerance test (GTT), the mice were fasted overnight and subsequently orally administered glucose (150 mg/mouse). Blood samples were obtained at 0, 30, 60, 90, and 120 min post-glucose administration, and glucose levels were immediately measured using a G400 green doctor blood glucose meter (Green Cross Medical Science Corp, Yongin-si, Republic of Korea) [24].

### 2.4. Enzyme-Linked Immunosorbent Assay

Serum tumor necrosis factor-alpha (TNF-α) levels were measured using an ELISA kit (Abcam, Cambridge, UK), following the manufacturer’s protocol. Duplicate samples from distinct lysates (*n* = 4/group) were used, which was repeated twice.

### 2.5. Histological Analysis

The liver and small intestine from each mouse of different groups were fixed in neutral formalin and embedded in paraffin blocks. Subsequently, tissue slides were stained with hematoxylin and eosin (H&E) or Sirius Red solution (Kyungpook National University Core lab, Daegu-si, Republic of Korea) [25]. Histological alterations were assessed under a light microscope [25].

### 2.6. Measurements of Serum Alanine Transaminase (ALT), Endotoxin, and Hepatic Triglyceride (TG) Levels

Serum levels of ALT and hepatic TG were measured using commercially available assay kits, such as the standard end-point colorimetric assay kit (BioVision, Milpitas, CA, USA, and Asan Co., Ltd., Gimpo, Republic of Korea, respectively). Additionally, serum endotoxin concentrations were measured using a commercial detection kit (endpoint LAL Chromogenic Endotoxin Quantitation Kit; Thermo Fisher Scientific, Waltham, MA, USA) [26,27].

### 2.7. Immunoblot Analysis

Tissues were homogenized using 1× radioimmunoprecipitation assay buffer. Lysates of specific tissues were separated using sodium dodecyl sulfate-polyacrylamide gel electrophoresis and transferred to nitrocellulose membranes. Subsequently, the nitrocellulose membranes were incubated with specific antibodies (Supplementary Table S1). Following incubation with the secondary antibodies, the images were detected using an ECL solution (Thermo Fisher). The band intensities of the immunoreactive target proteins were quantified relative to glyceraldehyde 3-phosphate dehydrogenase, which served as a loading control, through densitometry analysis using a chemiluminescence imaging system (FUSION SOLO S; Vilber, Collégien, France).

### 2.8. Statistical Analysis

Data are expressed as the mean ± standard error, and all statistical analyses were conducted using the SPSS/Windows 27.0 Statistical Package for the Social Sciences (SPSS Inc., Chicago, IL, USA). Student’s *t*-test was used to determine statistical significance (* *p* < 0.05, ** *p* < 0.01, and *** *p* < 0.001) [25].

## 3. Results

### 3.1. Metabolic Consequences in MASLD Mice

**Figure 2:**
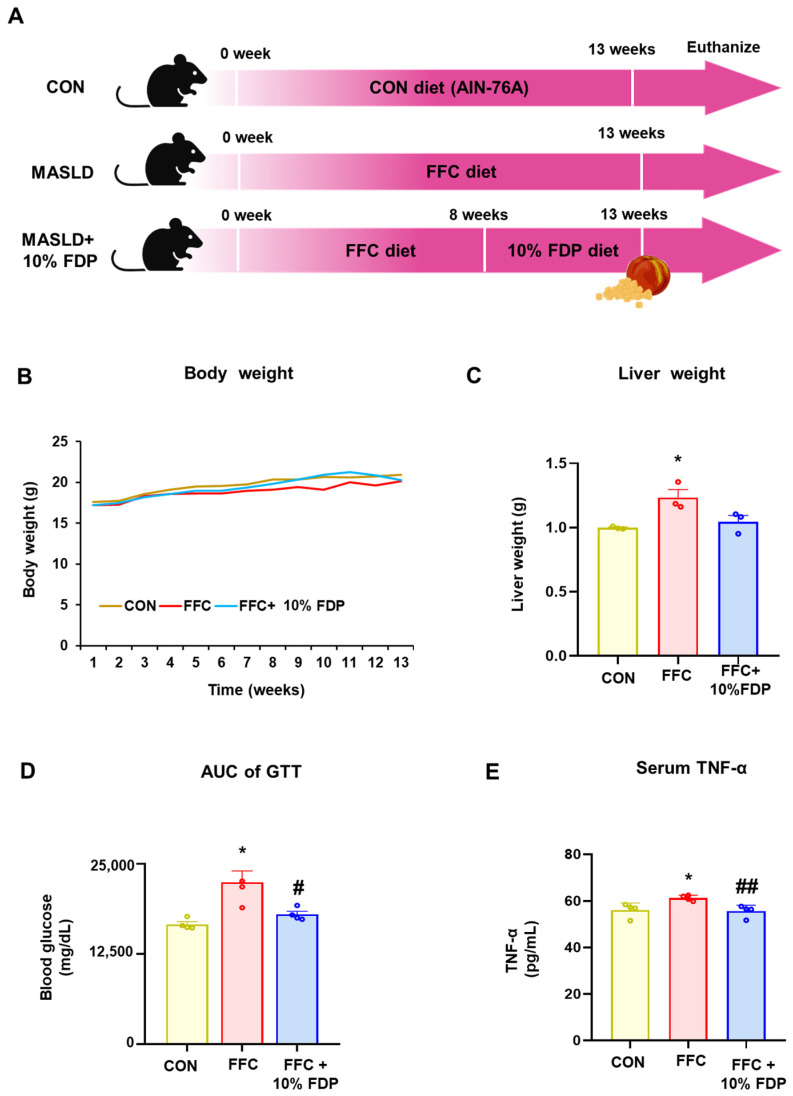
Effect of freeze-dried plum (FDP) diet on body weight, glucose metabolism, and inflammation markers in metabolic dysfunction-associated fatty liver disease (MASLD) mice. (**A**) Schematic representation to study the effects of plum diet against MASLD mice. (**B**) The representative body weight. (**C**) The representative liver weight. (**D**) Glucose tolerance testing of the area under the curve for MASLD mice. (**E**) Tumor necrosis factor-alpha levels are measured using enzyme-linked immunosorbent assay. * *p* < 0.05, between CON and high-fat, -fructose, and -cholesterol (FFC) groups; # *p* < 0.05, ## *p* < 0.01 between FFC and FFC + 10% FDP groups. The significance of mean values for each group is determined using Student’s *t*-test.

The hepatoprotective effect of 10% FDP against liver disease was assessed in MASLD mice fed an FFC diet (Figure 2A). There were no significant differences in body weight change (Figure 2B). Liver weight increased significantly in the FFC group compared to that in the control group, whereas it was reduced in the 10% FDP group (Figure 2C). The oral glucose tolerance test (OGTT) was performed to assess glucose tolerance in MASLD mice induced by FCC diets. Therefore, OGTT was increased in the FFC group compared to that in the control group but was reduced compared to normal values in the FFC + 10% FDP-treated group (Figure 2D). Enzyme-linked immunosorbent assay (ELISA) analysis revealed that MASLD mice exhibited increased serum TNF-α levels, which were significantly reduced through 10% FDP treatment (Figure 2E). Therefore, 10% FDP treatment prevented inflammation in the MASLD mouse models. In summary, we demonstrated that 10% FDP treatment reduces increased glucose tolerance and inflammation levels in an FFC-induced MASLD mouse model.

### 3.2. FDP Attenuated the Levels of Increased TG and Oxidative Stress Proteins in MASLD Mice

**Figure 3:**
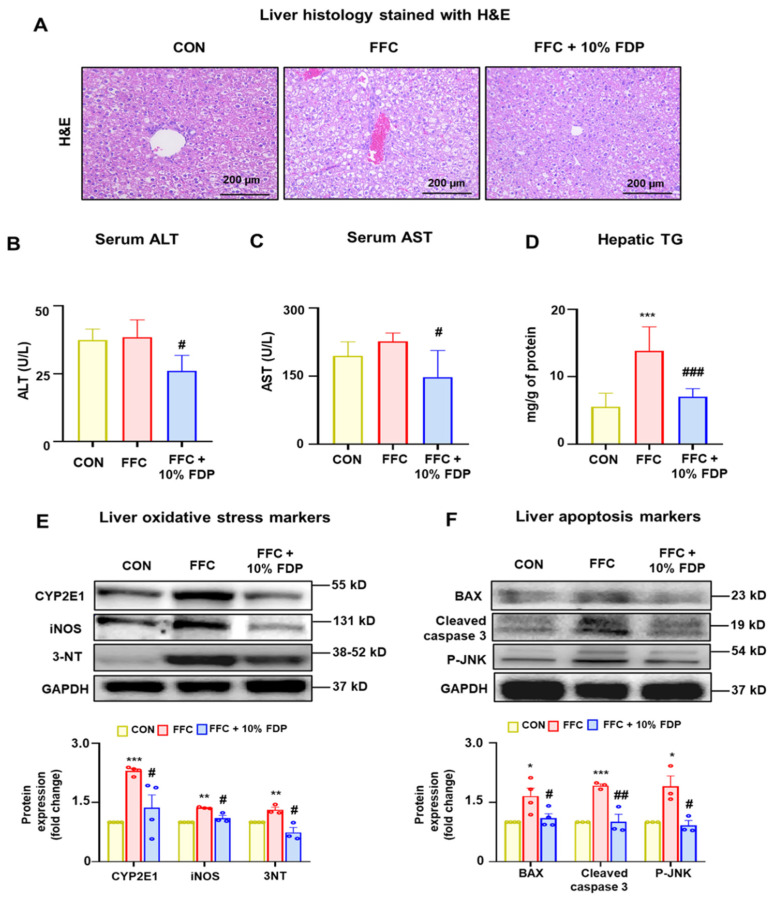
Freeze-dried plum (FDP) diet attenuated high-fat, -fructose, and -cholesterol (FFC)-induced liver injury in metabolic dysfunction-associated fatty liver disease mice. (**A**) Representative hematoxylin and eosin-stained liver sections for CON, FFC, FFC + 10% FDP group, as indicated. The levels of (**B**) serum alanine aminotransferase, (**C**) aspartate aminotransferase, and (**D**) hepatic triglycerides are presented. Immunoblot analyses for (**E**) oxidative stress markers (cytochrome P450 2E1, inducible nitric oxide synthase, and 3-nitrotyrosine) and (**F**) apoptosis markers (Bax, cleaved caspase 3, and phosphorylated c-Jun *N*-terminal kinase) for the indicated groups. Densitometric analysis of immunoblotting for each protein is demonstrated relative to the glyceraldehyde 3-phosphate dehydrogenase loading control. * *p* < 0.05, ** *p* < 0.01, and *** *p* < 0.001 between CON and FFC groups; # *p* < 0.05, ## *p* < 0.01, and ### *p* < 0.001 between FFC and FFC + 10%.

To determine the degree of liver damage in the MASLD mouse model, we assessed liver images and tissue staining. Liver imaging revealed damage in the FFC group compared with that in the control group. However, 10% FDP treatment alleviated these abnormalities in the liver (Supplementary Figure S1). Histological examination of H&E-stained slides revealed increased hepatic lipid droplets and inflammation in the FFC group relative to that in the control group, which was significantly reduced by dietary intervention with 10% FDP (Figure 3A). Additionally, serum levels of ALT and aspartate aminotransferase (AST) were significantly higher in the FFC group than that in the control group; however, these elevations were reduced by the 10% FDP dietary intervention (Figure 3B,C). Moreover, the hepatic TG levels were significantly increased in the FFC group, and the dietary intervention with 10% FDP significantly reduced the elevated TG levels (Figure 3D). Furthermore, liver oxidative stress markers (cytochrome P450 2E1 [CYP2E1], inducible nitric oxide synthase [iNOS], and 3-nitrotyrosine [3-NT]) and apoptotic markers (Bax, cleaved caspase 3, and phosphorylated c-Jun *N*-terminal kinase) were significantly increased in the FFC group. However, these levels were significantly reduced following dietary intervention with 10% FDP (Figure 3E,F). These findings indicate that FDP may offer protection against FFC-associated liver damage by reducing the elevated levels of oxidative stress and apoptosis markers.

### 3.3. FDP Attenuated the Increased Hepatic Fibrosis in MASLD Mice

**Figure 4:**
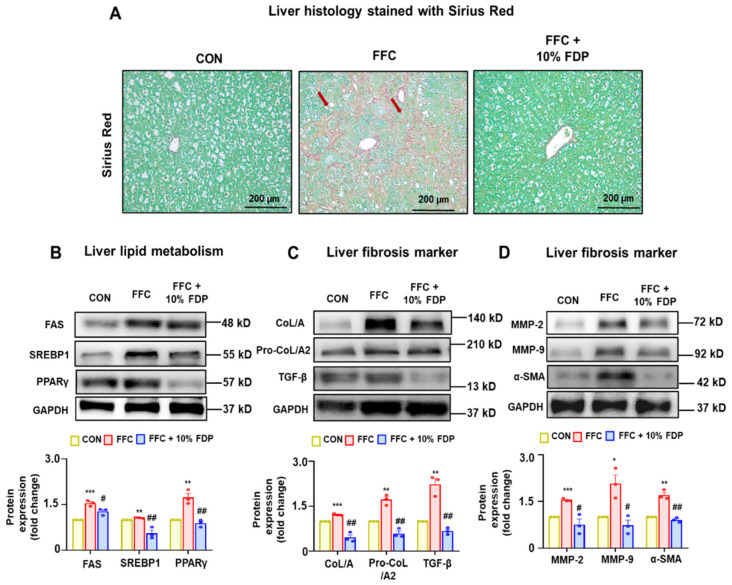
Freeze-dried plum (FDP) diet attenuated high-fat, -fructose, and -cholesterol (FFC)-induced liver fibrosis in metabolic dysfunction-associated fatty liver disease mice. (**A**) Representative Sirius Red-stained liver sections for CON, FFC, FFC + 10% FDP, as indicated. Immunoblot results for (**B**–**D**) various liver lipid metabolism markers (fatty acid synthase, sterol regulatory element-binding protein 1, and peroxisome proliferator-activated receptor gamma) or fibrosis markers (collagen type I alpha [COL/A], Pro-COL/A2, transforming growth factor beta, matrix metalloproteinase 2-, MMP-9, and alpha-smooth muscle actin) for the indicated groups. Densitometric analysis of immunoblotting for each protein is demonstrated relative to the glyceraldehyde 3-phosphate dehydrogenase loading control. * *p* < 0.05, ** *p* < 0.01, and *** *p* < 0.001 between CON and FFC groups; # *p* < 0.05, ## *p* < 0.01, between FFC and FFC + 10% FDP groups. The significance of mean values for each group is determined using Student’s *t*-test.

The protective effects of FDP against liver fibrosis were assessed using a MASLD mouse model. Sirius Red staining revealed larger regions of liver fibrosis in the FFC group than that in the control group, indicating that prolonged exposure to the FFC diet resulted in hepatic fibrosis in mice. However, reduced fibrotic areas were observed in the 10% FDP-treated groups compared to that in the control group (Figure 4A). Additionally, liver lipid metabolism (fatty acid synthase [FAS], sterol regulatory element-binding protein 1 [SREBP1], and peroxisome proliferator-activated receptor gamma) and fibrosis (collagen type I alpha [COL/A], PRO-COL/A2, transforming growth factor beta [TGF-β], matrix metalloproteinase [MMP]2, MMP9, and alpha-smooth muscle actin [α-SMA]) markers were increased in the FFC-treated group. However, these increases were significantly reduced by the dietary intervention with 10% FDP (Figure 4B–D). These results indicate that FDP exhibits a beneficial effect on FFC-associated liver fibrosis by suppressing the expression of fibrosis markers.

### 3.4. FDP Restored Gut Junctional Complex Proteins in MASLD Mice

**Figure 5:**
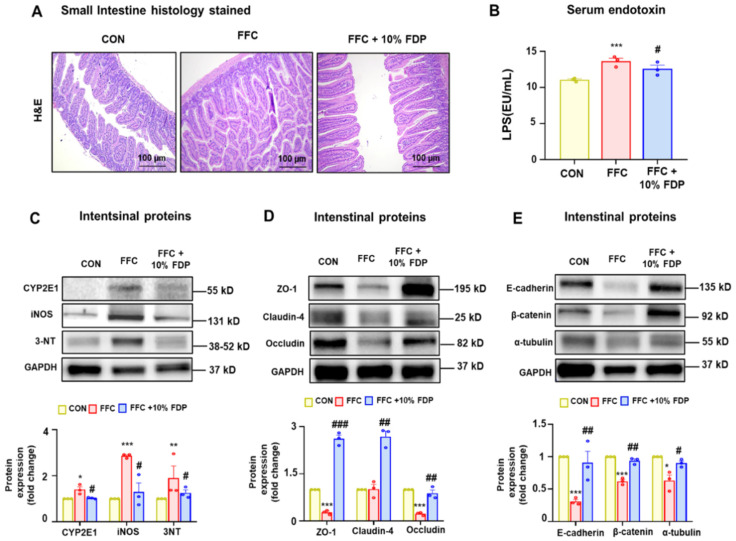
Freeze-dried plum (FDP) diet prevented high-fat, -fructose, and -cholesterol (FFC)-induced leaky gut in metabolic dysfunction-associated fatty liver disease mice. (**A**) Representative sections of the small intestine stained with hematoxylin and eosin for CON, FFC, FFC + 10% FDP, as indicated. (**B**) Serum endotoxin levels. (**C**) Levels of oxidative stress protein markers (cytochrome P450 2E1, inducible nitric oxide synthase, and 3-nitrotyrosine). (**D**) Levels of gut tight junction proteins (zonula occludens-1, claudin-4, and occludin) or (**E**) adherens junction proteins (E-cadherin, β-catenin, and α-tubulin). Densitometric analysis of immunoblotting for each protein is demonstrated relative to the glyceraldehyde 3-phosphate dehydrogenase loading control. * *p* < 0.05, ** *p* < 0.01, and *** *p* < 0.001 between CON and FFC groups; # *p* < 0.05, ## *p* < 0.01, and ### *p* < 0.001 between FFC and FFC + 10% FDP groups. The significance of mean values for each group is determined using Student’s *t*-test.

Recent studies have highlighted the association between the intestinal microbiome and non-alcoholic fatty liver disease (NAFLD). An imbalance in gut microbes can affect the progression of NAFLD by disrupting the gut–liver axis regulation [28,29,30]. Therefore, we confirmed gut damage and leaky gut owing to MASLD. H&E-stained histological analysis demonstrated that dietary intervention with 10% FDP enhanced the abnormal gut villus structure caused by the FFC diet (Figure 5A). Additionally, serum endotoxin levels were increased in the FFC group but this increase was significantly mitigated by the 10% FDP intervention (Figure 5B). Moreover, oxidative stress markers (CYP2E1, iNOS, and 3-NT) and junctional complex proteins (zonula occludens-1 [ZO-1], claudin-4, occludin, E-cadherin, β-catenin, and α-tubulin) in the small intestine were reduced in the FFC group but were restored in the 10% FDP-treated group (Figure 5C–E). In summary, in MASLD mice, FDP dietary intervention significantly reduced leaky gut and endotoxemia by increasing junctional complex proteins. This indicates the significance of plum dietary intervention against MASLD-mediated gut and liver damage and its role as a protective agent.

## 4. Discussion

MASLD is a chronic liver disease that causes various metabolic disorders, including hepatic steatosis, owing to high fructose intake and Western diet. This highlights the wide range of metabolic diseases and characteristics of all liver diseases [29,30]. Although research on MASLD has been increasing recently, there is one approved drug and limited treatment options, highlighting the urgent need for the development of novel drugs [31]. Additionally, a significant aspect of MASLD is the occurrence of leaky gut, alterations in the gut microbiota, and gut dysbiosis [4,32]. Gut dysbiosis is significantly implicated in the pathogenesis of MASLD through the gut–liver axis that connects the gut and liver. In mice fed a high-fat diet, the number of tight junctions is significantly reduced, resulting in gut leakage [33,34]. Lipopolysaccharides subsequently traverse the compromised gut barrier into the portal vein, where they activate Toll-like receptors and trigger the release of inflammatory cytokines, which cause liver damage and inflammation [35,36]. Moreover, owing to the limited treatment options and various health issues associated with MASLD, dietary interventions and lifestyle changes are essential.

Japanese plums (*P. salicina*) are highly effective natural antioxidants and anti-inflammatory agents in our diet owing to their high content of natural plant compounds, such as anthocyanins, flavonols, and phenolic acids, compared with other fruits [15,16,18,19,20,23]. Immature plum extract inhibits the growth of HepG2 hepatocarcinoma cells, exerts protective effects against liver damage by acting on antioxidant response elements [15], and exerts a protective effect on the intestine and liver in acute and chronic models of dextran sulfate sodium-induced colitis in mice [17]. Consuming plum juice helps regulate a cluster of pathways that are disrupted in obesity, thereby preventing obesity-related metabolic disorders and reducing the risk of cardiovascular disease [37]. Additionally, it has a preventive effect against inflammation and prevents bone loss owing to oophorectomy when consuming dried plums [38]. Therefore, we assessed the potential preventive effects of plums on liver damage and gut leakage in mice with MASLD induced by a Western-style rich FCC diet.

In this study, serum TNF-α levels were significantly increased in the MASLD model, whereas they were significantly reduced in the FDP dietary supplementation group, indicating that FDP has a significant protective effect on enhancing inflammation. Additionally, FDP protected against intestinal leakage of gut junction proteins, such as ZO-1, occludin, E-cadherin, and β-catenin, and inhibited oxidative stress markers, such as CYP2E1 and iNOS. Therefore, we demonstrated that FDP dietary intervention has a significant protective effect against leaky gut owing to MASLD.

The H&E staining revealed that MASLD mice exhibited increased levels of hepatic TG and fat accumulation in the liver that were mitigated by FDP treatment. Furthermore, FDP reduced the increased serum ALT and AST levels observed in MASLD mice. FDP inhibited the increase in hepatic lipid metabolism markers, such as FAS and SPEBP1, and oxidative stress markers, including CYP2E1, iNOS, and 3-NT. These findings indicate that FDP offers significant hepatoprotective benefits against liver damage in MASLD mice. Additionally, Sirius Red staining demonstrated a reduction in liver fibrosis following dietary intervention with FDP.

Moreover, Sirius Red and H&E staining indicated that liver fibrosis was reduced after dietary intervention with FDP. Specifically, FDP alleviated liver fibrosis by reducing the levels of COL/A, TGF-β, α-SMA, and MMP-2 in FFC diet-induced MASLD mice. In summary, this indicates that FDP intervention is significantly effective in alleviating leaky gut, liver fibrosis, and fatty liver disease caused by MASLD. Additionally, plum juice may play a significant role in preventing liver damage by modulating the gut–liver axis, a pathway that links gut microbiota and intestinal health with liver function, potentially reducing inflammation and oxidative stress in the liver (Figure 1).

## 5. Conclusions

In conclusion, this study demonstrated for the first time that plum consumption positively impacts liver health in a MASLD mouse model. Notably, it significantly inhibited the progression of hepatic fibrosis associated with fatty liver and leaky gut. This suggests that the antioxidant and anti-inflammatory properties of plums may have been the primary mechanisms behind this protective effect. The natural compounds in plums appeared to reduce liver damage by alleviating oxidative stress, modulating inflammatory responses, and improving intestinal barrier permeability to suppress inflammation caused by leaky gut. Given that MASLD is one of the chronic liver diseases with limited treatment options, plums can be highlighted as a promising natural-based therapeutic alternative in the current absence of effective treatments. Therefore, plums have the potential to be used as a novel approach in the treatment of liver diseases, and they also show promise as functional foods or natural therapeutic agents in the food and pharmaceutical industries. Further studies are needed to evaluate the clinical applicability of plums, paving the way for plum-based treatments to become effective therapies for MASLD patients.

## Data Availability

The data presented in this study are available on request from the corresponding author due to privacy restrictions.

## Supplementary Materials

The following supporting information can be downloaded at: https://www.mdpi.com/article/10.3390/nu16213760/s1; Figure S1: Effect of FDP dietary intervention on hepatic steatosis in normal or MASLD-induced mice; Table S1: The primary antibodies used in immunoblotting analyses.
