# Supplementary material for: Protective Effects of Plum on Liver and Gut Injury in Metabolic Dysfunction-Associated Fatty Liver Disease
Source: Nutrients. 2024 Nov 1;16(21):3760. doi: 10.3390/nu16213760 (PMC11548136; doi:10.3390/nu16213760)
Supplement: Supplementary file 1 [file nutrients-16-03760-s001.zip › nutrients-3277694-supplementary.pdf]

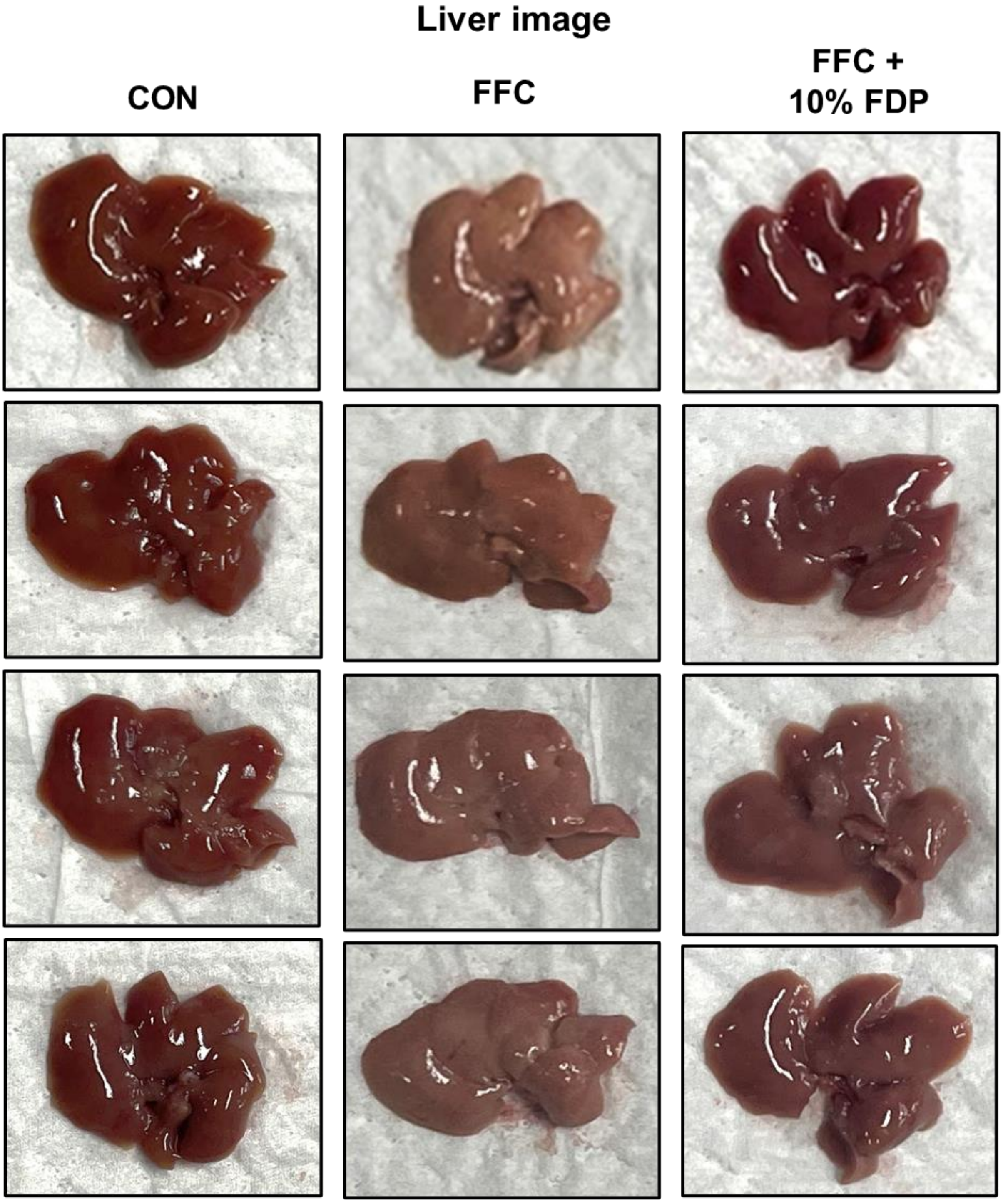


**Figure S1. Effect of FDP dietary intervention on hepatic steatosis in normal or MASLD-induced mice**. Representative photographs of liver tissues

**Table S1. The primary antibodies used in immunoblotting analyses.**

| | Antibody | Dilution Factor | Corporation | Clone |
|---|---|---|---|---|
| Primary antibody | **CYP2E1** | 1:5,000 | Abcam | Rabbit |
| | **iNOS** | 1:5,000 | Abcam | Rabbit |
| | **3NT** | 1:5,000 | Abcam | Mouse |
| | **FAS** | 1:1,000 | Santa Cruz | Mouse |
| | **PPARγ** | 1:1,000 | Santa Cruz | Mouse |
| | **SREBP-1** | 1:1,000 | Santa Cruz | Mouse |
| | **α-SMA** | 1:5,000 | Sigma-Aldrich | Mouse |
| | **MMP2** | 1:1,000 | Santa Cruz | Rabbit |
| | **MMP9** | 1:1,000 | Santa Cruz | Mouse |
| | **TGF-β** | 1:1,000 | Santa Cruz | Mouse |
| | **Collagen-1** | 1:1,000 | Santa Cruz | Mouse |
| | **Pro collagen-1** | 1:1,000 | Santa Cruz | Mouse |
| | **ZO-1** | 1:5,000 | Abcam | Mouse |
| | **Claudin-4** | 1:1,000 | Santa Cruz | Mouse |
| | **β-catenin** | 1:1,000 | Santa Cruz | Mouse |
| | **α-tubulin** | 1:1,000 | Santa Cruz | Mouse |
| | **E-cadherin** | 1:1,000 | Santa Cruz | Mouse |
| | **Bax** | 1:1,000 | Santa Cruz | Mouse |
| | **Occludin** | 1:1,000 | Santa Cruz | Mouse |
| | **Cleaved caspase 3** | 1:1,000 | Cell Signaling | Rabbit |
| | **P-JNK** | 1:1,000 | Santa Cruz | Mouse |
| | **JNK** | 1:1,000 | Santa Cruz | Mouse |
| | **GAPDH** | 1:1,000 | Santa Cruz | Mouse |
